# Clinical metagenomics of bone and joint infections: a proof of concept study

**DOI:** 10.1038/s41598-017-07546-5

**Published:** 2017-08-10

**Authors:** Etienne Ruppé, Vladimir Lazarevic, Myriam Girard, William Mouton, Tristan Ferry, Frédéric Laurent, Jacques Schrenzel

**Affiliations:** 1 Genomic Research Laboratory, Service of Infectious Diseases, Geneva University Hospitals, rue Gabrielle-Perret-Gentil 4, 1205 Geneva, Switzerland; 2 Centre International de Recherche en Infectiologie, INSERM U1111, Pathogenesis of staphylococcal infections, University of Lyon 1, Lyon, France; 3 Department of Clinical Microbiology, Northern Hospital Group, Hospices Civils de Lyon, Lyon, France; 4 Infectious Diseases Department, Northern Hospital Group, Hospices Civils de Lyon, Lyon, France; 5 Bacteriology Laboratory, Service of Laboratory Medicine, Department of Genetics and Laboratory Medicine, Geneva University Hospitals, 4 rue Gabrielle-Perret-Gentil, 1205 Geneva, Switzerland

## Abstract

Bone and joint infections (BJI) are severe infections that require a tailored and protracted antibiotic treatment. Yet, the diagnostic based on culturing samples lacks sensitivity, especially for hardly culturable bacteria. Metagenomic sequencing could potentially address those limitations. Here, we assessed the performances of metagenomic sequencing on 24 BJI samples for the identification of pathogens and the prediction of their antibiotic susceptibility. For monomicrobial samples in culture (n = 8), the presence of the pathogen was confirmed by metagenomics in all cases. For polymicrobial samples (n = 16), 32/55 bacteria (58.2%) were found at the species level (and 41/55 [74.5%] at the genus level). Conversely, 273 bacteria not found in culture were identified, 182 being possible pathogens and 91 contaminants. A correct antibiotic susceptibility could be inferred in 94.1% and 76.5% cases for monomicrobial and polymicrobial samples, respectively. Altogether, we found that clinical metagenomics applied to BJI samples is a potential tool to support conventional culture.

## Introduction

### Context

Bone and joint infections (BJI) are severe infections that affect a growing number of patients^1^. Along with the surgical intervention, the microbiological diagnosis is a keystone of the management of BJI in (*i*) identifying the bacteria causing the infection and (*ii*) assessing their susceptibility to antibiotics. Currently, this is achieved by culturing surgical samples on various media and conditions, together with a long time of incubation to recover fastidiously-growing bacteria that can be involved in BJI. Still, some bacteria will not grow under these conditions because of extreme oxygen sensitivity, a prior antibiotic intake or metabolic issues (*e*.*g*. quiescent bacteria in chronic infections). Consequently, the antibiotic treatment may not span all the bacteria involved in the infection, which can favour the relapse and the need for a new surgery.

Clinical metagenomics refers to the concept of sequencing all the DNA (*i*.*e*. all the genomes) present in a clinical sample with the purpose of identifying pathogens and inferring their antibiotic susceptibility pattern^2^. This new, culture-independent method takes advantages of the thrilling development of next-generation sequencing (NGS) technologies since the mid-2000s. The NGS platforms typically yield thousands to millions of reads (sequences of size ranging from 100 bp to a few kbp), which virtually enables to recover the sequences of all the genes present in the sample, yet in a disorganised fashion. Substantial bio-informatics efforts are thereby needed to re-construct and re-order the original sequences in genomes, and are referred to as the assembly process. Hence, various information such as the taxonomic identification of the present species, antibiotic resistance determinants (ARDs), mutations (as compared to a reference genome or sequence), single nucleotide variants (SNVs, for clonality assessment) and virulence genes can be determined.

Clinical metagenomics is an emerging field in medicine. So far, a few attempts to use metagenomics on clinical samples have been performed (on urines^3, 4^, cerebrospinal fluid or brain biopsy^5, 6^, blood^7^ and skin granuloma^8^) likely because of the high price of metagenomics and the complexity of the management of sequence data for clinical microbiologists. To the best of our knowledge, metagenomics has never been applied to BJI samples.

As for the inference of antibiotic susceptibility testing from the genomic information, a few studies focusing on *Escherichia coli*, *Klebsiella pneumoniae*, *Mycobacterium tuberculosis* and *Staphylococcus aureus* have constantly shown excellent correlations between the analysis of the genomic content of antibiotic resistance determinants (ARDs) and the phenotype^9–15^ while performances were not as good for *Pseudomonas aeruginosa* ^16^. Furthermore in metagenomic data, the possible presence of multiple pathogens raises the issue of linking ARDs to their original host in order to infer its antibiotic susceptibility pattern^3^. So far no method has been proposed to address this question.

Applying metagenomics in the context of BJI is thus seducing in that (*i*) there is no limit in the number of species and ARDs that could be detected (as opposed to PCR-based methods which detect targeted ARDs), (*ii*) unculturable bacteria, fastidious growers (such as *Propionibacterium* sp.) or bacteria altered by prior antibiotic use would be recovered, and (*iii*) the antibiotic susceptibility inference would benefit from both the detection of ARDs (such as *mecA*, *qnr*, *dfr*, *erm*, *etc*.) and the identification of mutations leading to resistance to key antibiotics used in BJI. Here, our main objective is to assess the performances of clinical metagenomics in BJI in terms of pathogen identification and inference of antibiotic susceptibility, as compared with conventional microbiology (gold standard).

## Results

### DNA extraction

We first extracted the samples for which the quantity of material exceeded or was equal to 1 mL (n = 77). We recovered more than 1 pg/µL bacterial DNA mostly for samples that had grown at >100 CFUs (Extended Data Fig. 1, panel A), while the concentration of human DNA did not correlate with the bacterial load (Extended Data Fig. 1, panel B). The remaining samples (<1 mL material), that had grown at least 100 CFUs (n = 25, see Extended Data), were submitted to extraction. In total, the DNA of 104 samples was extracted, from which 24 met our internal requirements for being sequenced (*i*.*e*. contained at least 1 pg/µL bacterial DNA, and less than 99% human DNA, Supplementary Table 1). Other samples could not be sequenced because of the low bacterial DNA concentration and/or a high human DNA concentration (Supplementary Table 1). These 24 samples were obtained from 14 patients (Table 1). All throughout the manuscript, we will refer as monomicrobial (n = 8) and polymicrobial (n = 16) samples which respectively yielded one and more than one bacterial species in culture.

**Figure 1:**
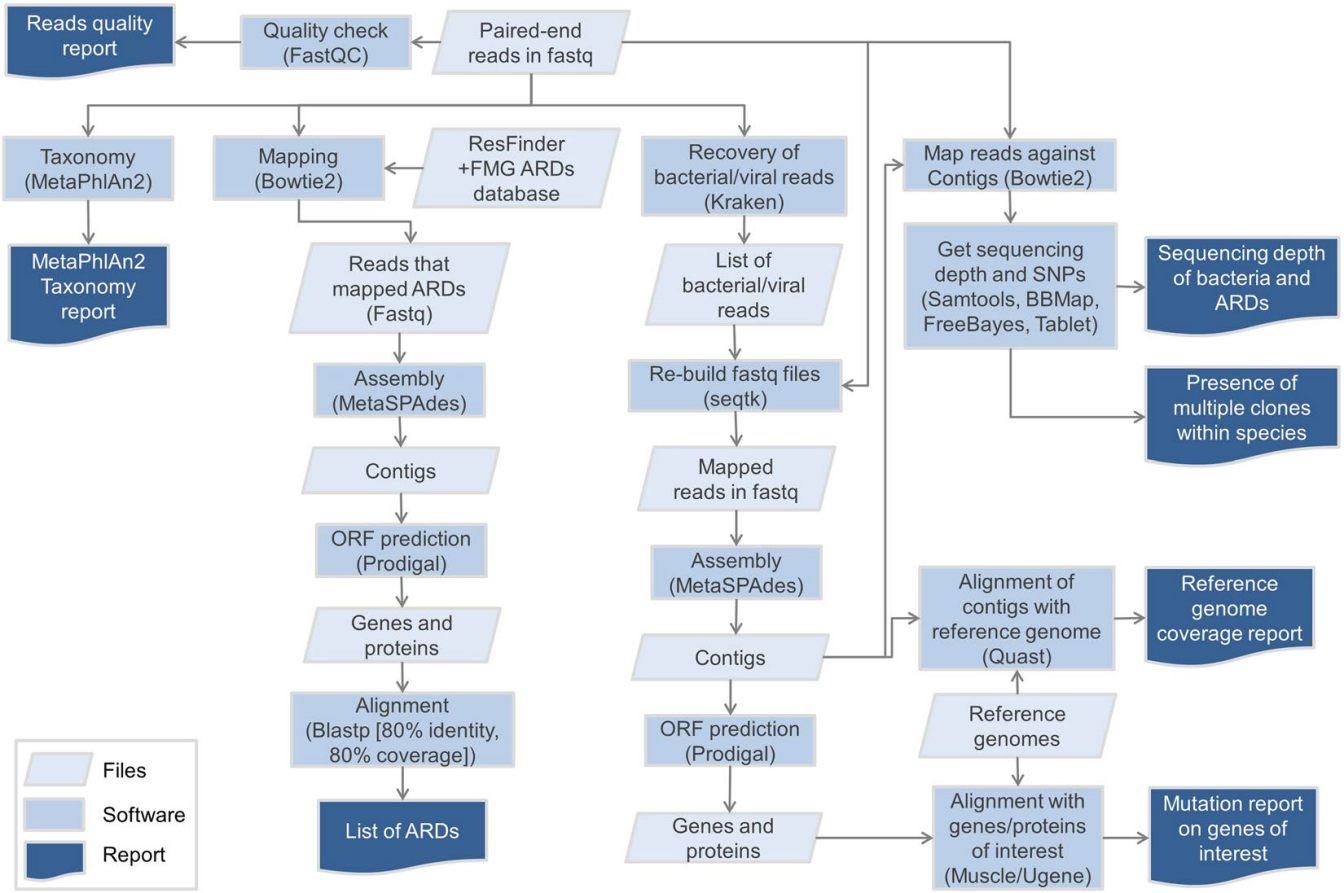
Bioinformatic analysis performed in this study. ARDs: antibiotic resistance determinants. Fastq: format for the files that embeds the read sequences and their per-base quality score. FMG: functional metagenomics; ARDs: antibiotic resistance determinants; ORF: open reading frame.

**Table 1:** Characteristics of the 14 patients for whom 24 samples were sequenced.

Patient	Samples	Age	Gender	ASA score	Body mass index	Post-operative infection (type of surgery)	Delay between surgery and infection	Body site	Material involved
A	2, 66, 28	51	M	2	30.4	Yes (material)	<1 month	Ankle	Osteosynthesis
B	4, 140	50	F	2	39.8	No	NA	Clavicle	None
C	19, 103, 104	54	M	2	24.1	Yes (material)	<1 month	Toe	Osteosynthesis
D	110	66	M	2	29.4	Yes (material)	Between 1 and 3 months	Tibia	Osteosynthesis
E	42	61	F	3	50.6	Yes (material)	<1 month	Knee	Total knee prothesis
F	46	63	M	2	18.0	Yes (material)	<1 month	Mandible	Osteosynthesis
G	59, 117, 136	69	M	2	25.5	Yes (bone resection)	NA	Tibia	None
H	90, 158	64	F	2	21.2	No	NA	Sacrum	None
I	108, 181	86	F	2	26.7	Yes (material)	Between 1 and 3 months	Knee	Total knee prothesis
J	121, 172	50	F	1	24.2	No	NA	Tibia	None
K	128	86	F	2	30.1	No	>3 months	Knee	Osteosynthesis
L	171	51	M	1	25.6	Yes (material)	>3 months	Tibia	Osteosynthesis
M	178	87	F	3	26.1	Yes (material)	<1 month	Knee	Total knee prothesis
N	184	60	M	3	34.3	No	NA	Greater trochanter and ischium	None

ASA: American Society of Anesthesiologists.

### Bioinformatics

The bioinformatic pipeline is depicted in the Fig. 1. After trimming, we obtained a mean number of 20,092,168 paired reads per sample (range 8,256,850–29,099,374, Supplementary Table 2). With the Kraken classifier, the mean rate of classified reads (as bacteria, archea or virus) was 27.9% (range 1.8–85.7, Supplementary Table 2). Of note, the classification rate was correlated to the proportion of bacterial DNA as found by qPCR (Pearson’s correlation test, p < 0.001, cor = 0.70, Extended Data Fig. 2). The assembly of the classified reads with metaSPAdes yielded a mean number of contigs of 10,444 (range 3,087–18,513, Supplementary Table 2), for a mean total number of base pairs of 8.3 M (range 2.9M–16.5 M, Supplementary Table 2). The total number of base pairs of contigs was higher in polymicrobial samples than in the monomicrobial ones (respectively 9.7 M vs. 5.5 M, t test p < 0.05, Extended Data Fig. 3). The mean size of the contigs was 805 bp (median 369 bp, maximum 445,300 bp, Extended Data Fig. 4 panel A). It was higher in polymicrobial samples than in monomicrobial samples (respectively 865 bp vs 654 bp, p < 0.001, Extended Data Fig. 4 panel B). Besides, the genome coverage of the bacteria found in culture was higher in monomicrobial samples than in polymicrobial samples (respectively 78.7% [98.8% when not considering the sample 46 which is actually a polymicrobial infection according to metagenomic findings] vs. 22.6%, p < 0.001, Extended Data Fig. 3 panel F).

**Figure 2:**
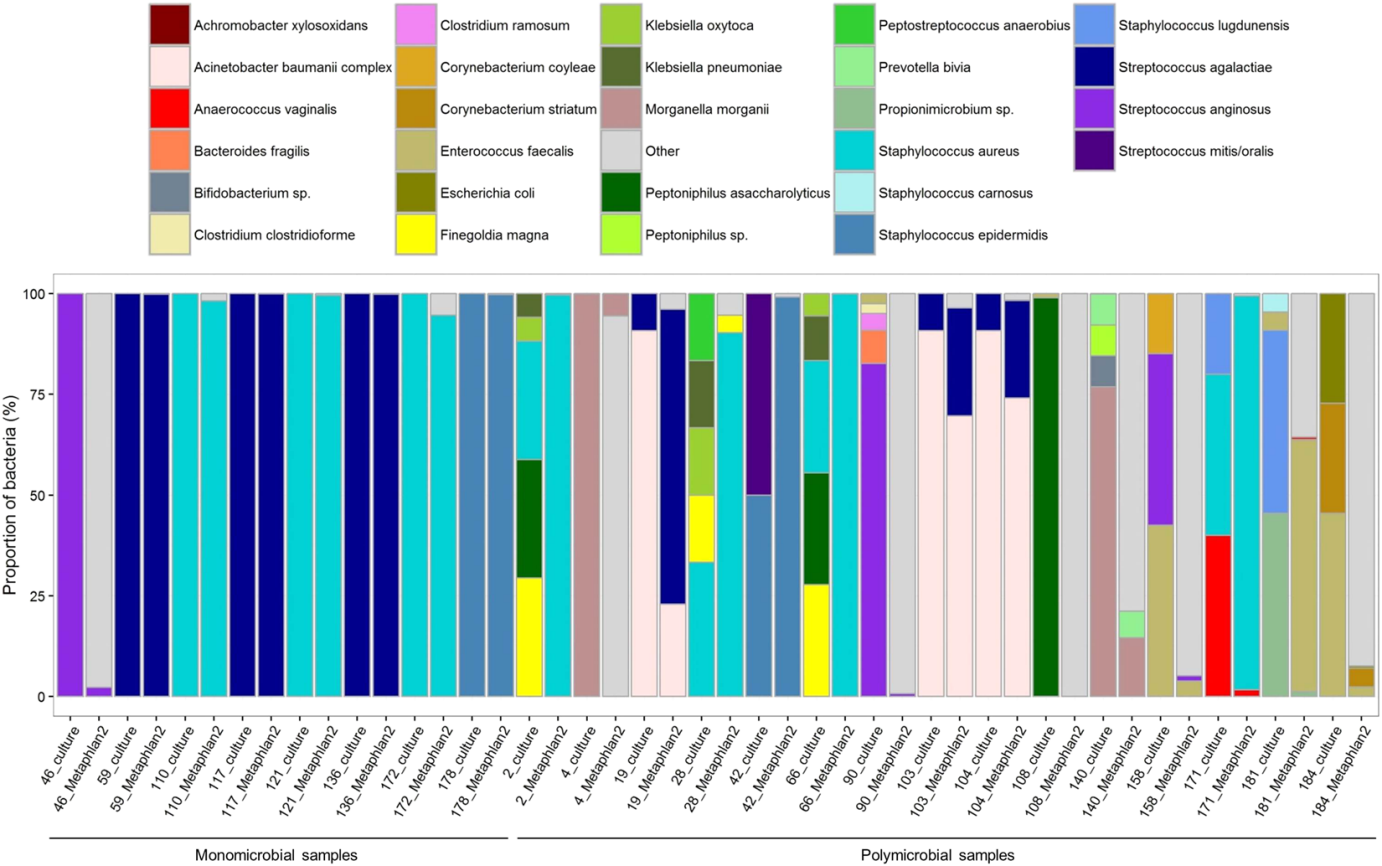
Proportions of the species recovered in culture and from reads (using MetaPhlAn2^24^).

**Figure 3:**
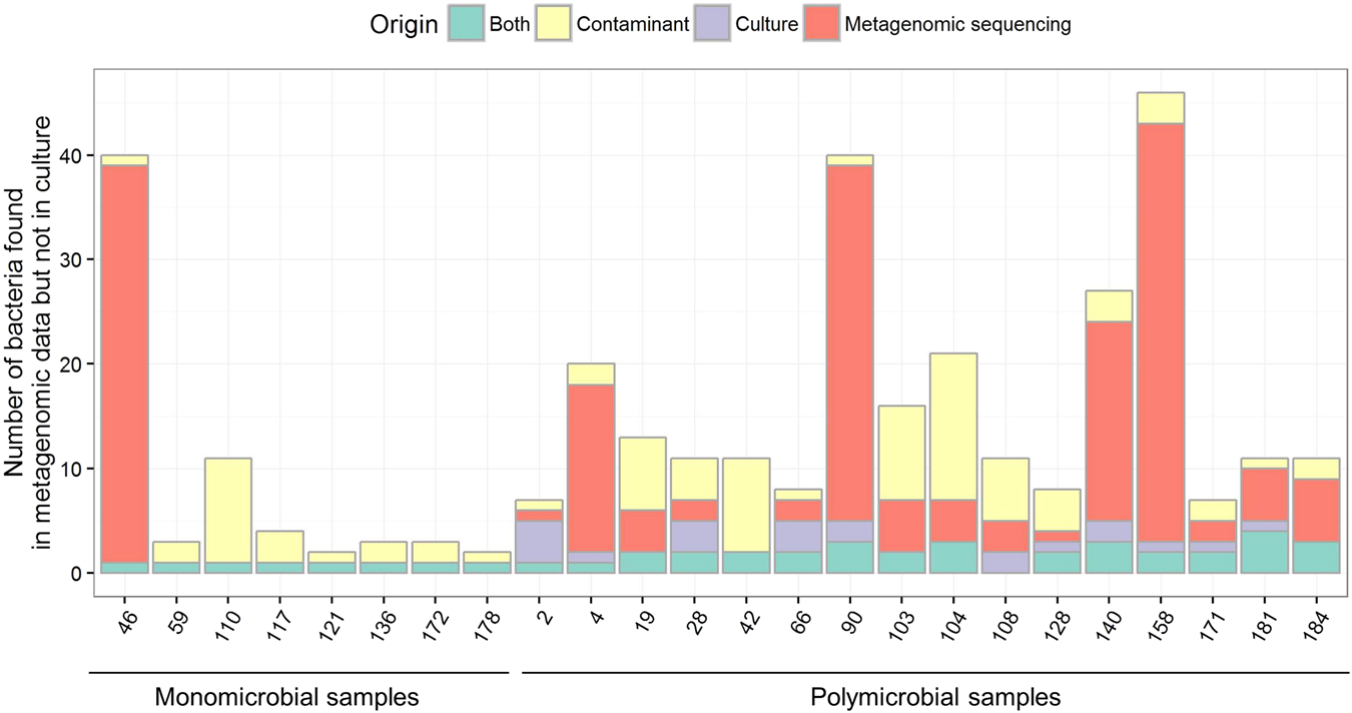
Distribution of the number of species found both in culture and metagenomic sequencing, only in culture and only metagenomic sequencing (in this case, putative pathogenic species and contaminants/misclassifications are depicted apart). All species identified by MetaPhlAn2 were considered (not only those above 0.1% abundance).

**Figure 4:**
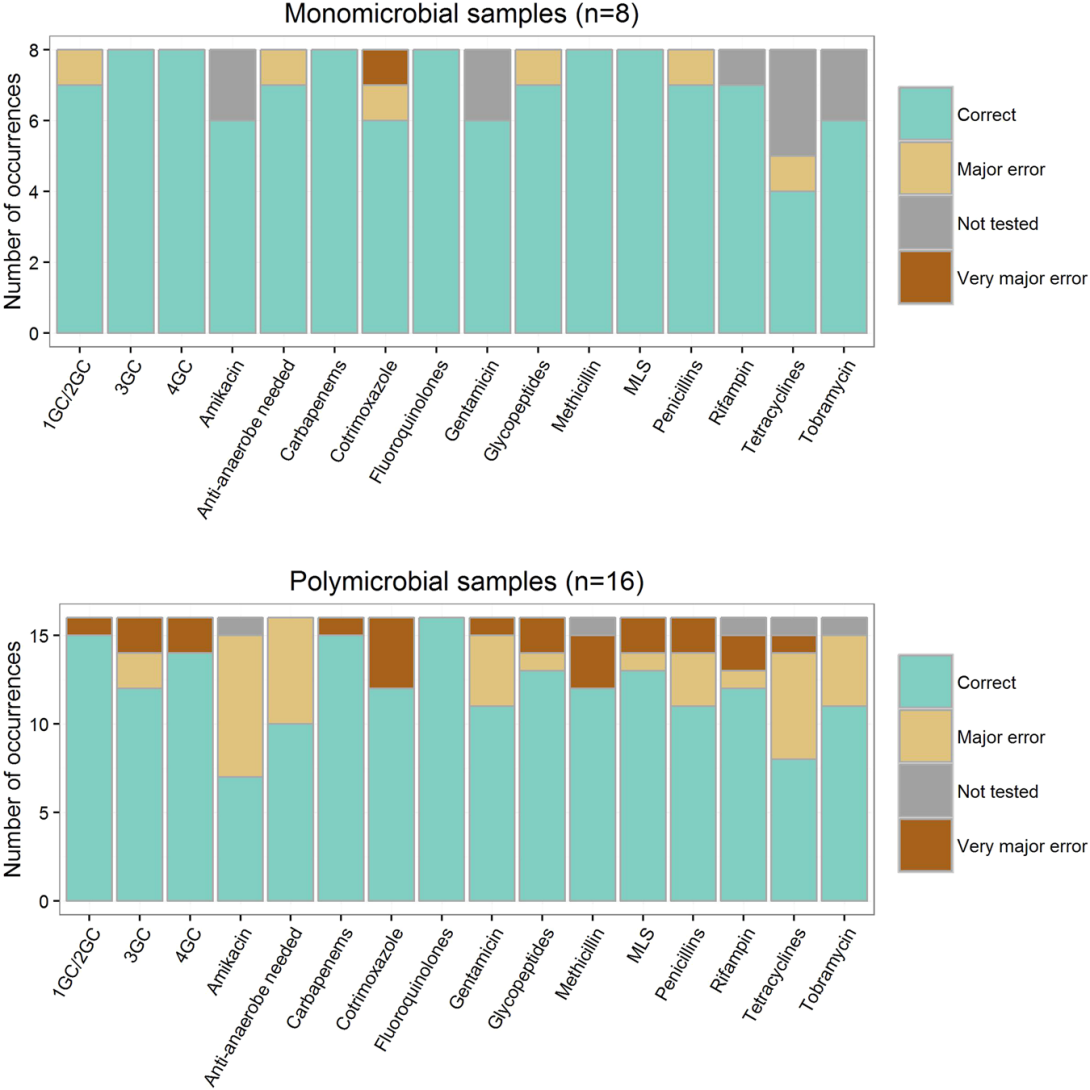
Antibiotic susceptibility inference from metagenomic data compared to culture and conventional antibiotic susceptibility testing (gold standard). 1GC/2GC, 3GC, 4GC: 1^st^, 2^nd^, 3^rd^ and 4^th^ generation cephalosporins, respectively. MLS: macrolides, lincosamides, streptogramines. Correct: metagenomic result consistent with the result given by conventional methods. Very major error: metagenomic data did not predict antibiotic resistance while at least one bacteria identified by conventional methods was resistant to this antibiotic. Major error: metagenomic data predicted antibiotic resistance while all the bacteria identified by conventional methods were susceptible. Not tested: no molecule from the antibiotic class was tested with conventional methods.

### Identification of the pathogens

In monomicrobial samples (n = 8, Table 2), the sensitivity of metagenomic sequencing was 100% (8/8) at both genus and species levels. The pathogens identified by culture were found by MetaPhlAn2 at very high relative abundances (over 94.6%) with the exception of sample 46 in which *Streptococcus anginosus* was found at a relative abundance of 2.2% (Fig. 2). In polymicrobial samples (n = 16, Table 2

**Table 2:** Results of the culture and metagenomic sequencing of the 24 samples analysed in this study.

Patient	Sample number	Monomicrobial or polymicrobial	Culture (proportion in %)	Species identified in metagenomic sequencing (≥0.1% abundance)
A	2	Polymicrobial	*Staphylococcus aureus* (29.4), *Klebsiella pneumoniae* (5.9), *Klebsiella oxytoca* (5.9), *Peptoniphilus asaccharolyticus* (29.4), *Finegoldia magna* (29.4)	*Staphylococcus aureus* (99.6)
A	28	Polymicrobial	*Staphylococcus aureus* (33.3), *Klebsiella pneumoniae* (16.7), *Klebsiella oxytoca* (16.7), *Finegoldia magna* (16.7), *Peptostreptococcus anaerobius* (16.7)	*Staphylococcus aureus* (90.3), *Finegoldia magna* (4.3), *Peptoniphilus harei* (3.7), *Propionibacterium acnes* (0.9)
A	66	Polymicrobial	*Staphylococcus aureus* (27.8), *Klebsiella pneumoniae* (11.1), *Klebsiella oxytoca* (5.6), *Finegoldia magna* (27.8), *Peptoniphilus asaccharolyticus* (27.8)	*Staphylococcus aureus* (99.9)
B	4	Polymicrobial	*Morganella morganii* (99.9), *Streptococcus anginosus* (0.1)	*Morganella morganii* (5.6), *Propionibacterium propionicum* (71.5), *Bacteroides fragilis* (14.2), *Prevotella bivia* (4.1), *Atopobium rimae* (3.0), *Parvimonas unclassified* (1.0), *Parvimonas micra* (0.2), *Prevotella buccalis* (0.1)
B	140	Polymicrobial	*Morganella morganii* (76.9), *Streptococcus anginosus* (0.1), *Prevotella bivia* (7.7), *Bifidobacterium* (7.7), *Peptoniphilus* (7.7)	*Morganella morganii* (14.6), *Prevotella bivia* (6.6), *Propionibacterium propionicum* (43.2), *Bacteroides fragilis* (24.4), *Atopobium rimae* (8.3), *Parvimonas* unclassified (1.2), *Dermabacter* sp HFH0086 (1.0), *Parvimonas micra* (0.3), *Anaeroglobus geminatus* (0.1), *Proteus mirabilis* (0.1)
C	19	Polymicrobial	*Acinetobacter baumanii complex* (90.8), *Streptococcus agalactiae* (9.2)	*Acinetobacter baumanii* complex (22.1), *Streptococcus agalactiae* (73.1), *Finegoldia magna* (1.7), *Acinetobacter/pittii/calcoaceticus nosocomialis* (0.9), *Corynebacterium resistens* (0.9), *Helcococcus kunzii* (0.7), *Advenella kashmirensis* (0.3), *Propionibacterium acnes* (0.2), *Achromobacter unclassified* (0.1), *Achromobacter piechaudii* (0.1)
C	103	Polymicrobial	*Acinetobacter baumanii complex* (90.8), *Streptococcus agalactiae* (9.2)	*Acinetobacter baumanii* complex (66.9), *Streptococcus agalactiae* (26.9), *Acinetobacter/pittii/calcoaceticus nosocomialis* (2.7), *Finegoldia magna* (1.5), *Corynebacterium resistens* (0.4), *Staphylococcus simulans* (0.4), *Propionibacterium acnes* (0.3), *Staphylococcus lugdunensis* (0.2), *Achromobacter* unclassified (0.1), *Helcococcus kunzii* (0.1), *Bordetella* unclassified (0.1), *Advenella kashmirensis* (0.1)
C	104	Polymicrobial	*Acinetobacter baumanii complex* (90.8), *Streptococcus agalactiae* (9.2), *Achromobacter xylosoxidans* (0.0)	*Acinetobacter baumanii* complex (70.1), *Streptococcus agalactiae* (24.2), *Acinetobacter/pittii/calcoaceticus nosocomialis* (3.9), *Corynebacterium resistens* (1.1), *Propionibacterium acnes* (0.2), *Achromobacter* unclassified (0.1), *Bordetella* unclassified (0.1), *Enhydrobacter aerosaccus* (0.1)
D	110	Monomicrobial	*Staphylococcus aureus* (100.0)	*Staphylococcus aureus* (98.2), *Propionibacterium acnes* (1.7)
E	42	Polymicrobial	*Staphylococcus epidermidis* (50.0), *Streptococcus mitis/oralis* (50.0)	*Staphylococcus epidermidis* (99.1), *Propionibacterium acnes* (0.4)
F	46	Monomicrobial	*Streptococcus anginosus* (100.0)	*Streptococcus anginosus* (2.2), *Mogibacterium* sp CM50 (34.5), *Olsenella uli* (13.8), *Atopobium* sp oral taxon 199 (13.0), *Peptostreptococcus stomatis* (6.9), *Parvimonas* unclassified (6.7), *Fretibacterium fastidiosum* (6.3), *Pseudoramibacter alactolyticus* (5.6), *Slackia* unclassified (3.2), *Slackia exigua* (2.2), *Alloprevotella tannerae* (2.0), *Prevotella oris* (0.8), *Eubacterium infirmum* (0.5), *Treponema maltophilum* (0.4), *Parvimonas micra* (0.3), *Prevotella baroniae* (0.2), *Treponema socranskii* (0.2), *Bacteroidetes bacterium* oral taxon 272 (0.2), *Dialister invisus* (0.2), *Prevotella denticola* (0.2), *Tannerella forsythia* (0.1), *Porphyromonas uenonis* (0.1), *Treponema vincentii* (0.1), *Treponema denticola* (0.1), *Peptoniphilus lacrimalis* (0.1)
G	59	Monomicrobial	*Streptococcus agalactiae* (100.0)	*Streptococcus agalactiae* (99.9)
G	117	Monomicrobial	*Streptococcus agalactiae* (100.0)	*Streptococcus agalactiae* (99.9), *Rickettsia japonica* (0.1)
G	136	Monomicrobial	*Streptococcus agalactiae* (100.0)	*Streptococcus agalactiae* (99.8), *Propionibacterium acnes* (0.1)
H	90	Polymicrobial	*Streptococcus anginosus* (82.6), *Enterococcus faecalis* (2.5), *Bacteroides fragilis* (8.3), *Clostridium ramosum* (4.1), *Clostridium clostridioforme* (2.5)	*Streptococcus anginosus* (0.6), *Olsenella* sp oral taxon 809 (55.9), *Pseudoramibacter alactolyticus* (9.6), *Atopobium* sp oral taxon 199 (8.4), *Eggerthella* unclassified (6.6), *Slackia* unclassified (5.8), *Mogibacterium* sp CM50 (5.3), *Slackia exigua* (1.8), *Peptoniphilus* sp oral taxon 375 (1.6), *Anaerococcus lactolyticus* (1.0), *Peptoniphilus harei* (0.6), *Finegoldia magna* (0.5), *Parvimonas* unclassified (0.4), *Peptoniphilus lacrimalis* (0.4), *Olsenella uli* (0.3), *Eggerthella lenta* (0.3), *Parvimonas micra* (0.1), *Porphyromonas asaccharolytica* (0.1), *Actinomyces europaeus* (0.1), *Actinomyces turicensis* (0.1), *Coriobacteriaceae bacterium* BV3Ac1 (0.1), *Porphyromonas somerae* (0.1), *Subdoligranulum* unclassified (0.1)
H	158	Polymicrobial	*Enterococcus faecalis* (42.6), *Streptococcus anginosus* (42.6), *Corynebacterium coyleae* (14.9)	*Enterococcus faecalis* (3.8), *Streptococcus anginosus* (1.3), *Olsenella* sp oral taxon 809 (69.8), *Mogibacterium* sp CM50 (6.5), *Slackia* unclassified (3.8), *Peptoniphilus harei* (1.6), *Atopobium* sp oral taxon 199 (1.5), *Finegoldia magna* (1.4), *Pseudoramibacter alactolyticus* (1.2), *Anaerococcus lactolyticus* (1.2), *Peptoniphilus* sp oral taxon 375 (1.1), *Slackia exigua* (1.0), *Porphyromonas asaccharolytica* (0.8), *Actinomyces turicensis* (0.8), *Subdoligranulum* unclassified (0.7), *Peptoniphilus lacrimalis* (0.7), *Eggerthella unclassified* (0.4), *Olsenella uli* (0.4), *Anaerococcus vaginalis* (0.2), *Lachnospiraceae bacterium* 5 1 57FAA (0.2), *Dialister invisus* (0.2), *Clostridium clostridioforme* (0.2), *Actinomyces europaeus* (0.2), *Porphyromonas somerae* (0.2), *Clostridiales bacterium* BV3C26 (0.1), *Anaerococcus obesiensis* (0.1), *Parvimonas* unclassified (0.1), *Helcococcus kunzii* (0.1), *Prevotella timonensis* (0.1), *Facklamia hominis* (0.1), *Eggerthella lenta* (0.1)
I	108	Polymicrobial	*Enterococcus faecalis* (1.0), *Peptoniphilus asaccharolyticus* (99.0)	*Peptoniphilus harei* (98.7), *Propionibacterium acnes* (0.7), *Streptococcus agalactiae* (0.1), *Deinococcus* unclassified (0.1), *Acinetobacter* unclassified (0.1)
I	181	Polymicrobial	*Enterococcus faecalis* (4.5), *Staphylococcus carnosus* (4.5), *Staphylococcus lugdunensis* (45.5), *Propionimicrobium*, 45.5, *Anaerococcus vaginalis* (0.0)	*Enterococcus faecalis* (62.5), *Propionimicrobium* (1.2), *Anaerococcus vaginalis* (0.5), *Peptoniphilus harei* (20.7), *Actinomyces neuii* (7.5), *Finegoldia magna* (7.1), *Anaerococcus obesiensis* (0.2), *Propionibacterium acnes* (0.1)
J	121	Monomicrobial	*Staphylococcus aureus* (100.0),	*Staphylococcus aureus* (99.6), *Propionibacterium acnes* (0.4)
J	172	Monomicrobial	*Staphylococcus aureus* (100.0),	*Staphylococcus aureus* (94.6), *Micrococcus luteus* (3.1), *Propionibacterium acnes* (2.2)
K	128	Polymicrobial	*Proteus mirabilis* (*NA*), *Klebsiella oxytoca* (*NA*), *Pseudomonas aeruginosa* (*NA*)	*Klebsiella oxytoca* (74.2), *Pseudomonas aeruginosa* (0.8), *Klebsiella* unclassified (23.5), *Rothia mucilaginosa* (0.3), *Pseudomonas* unclassified (0.3), *Propionibacterium acnes* (0.2)
L	171	Polymicrobial	*Staphylococcus aureus* (40.0), *Staphylococcus lugdunensis* (20.0), *Anaerococcus vaginalis* (40.0)	*Staphylococcus aureus* (97.8), *Anaerococcus vaginalis* (1.6), *Propionibacterium acnes* (0.5), *Bartonella* unclassified (0.1)
M	178	Monomicrobial	*Staphylococcus epidermidis* (100.0)	*Staphylococcus epidermidis* (99.7), *Propionibacterium acnes* (0.2)
N	184	Polymicrobial	*Escherichia coli* (27.3), *Enterococcus faecalis* (45.5), *Corynebacterium striatum* (27.3)	*Enterococcus faecalis* (2.3), *Corynebacterium striatum* (4.7), *Finegoldia magna* (53.6), *Dermabacter* sp HFH0086 (13.7), *Peptoniphilus harei* (10.3), *Varibaculum cambriense* (8.6), *Anaerococcus vaginalis* (2.3), *Propionibacterium acnes* (2.0), *Anaerococcus obesiensis* (0.9), *Escherichia* unclassified (0.5), *Corynebacterium pyruviciproducens* (0.1)

NA: not available.

), the sensitivity of metagenomic sequencing was 58.2% (32/55) at the species level. At the genus level, the sensitivity increased to 74.5% (41/55). Besides, metagenomic sequencing could identify all bacteria found by culture in a given sample in 11/24 (45.8%) samples at the species level, including 3/16 (18.8%) for polymicrobial infections. At the genus level, 15/24 (62.5%) samples were in agreement with cultures, including 7/16 (43.8%) samples with polymicrobial infections.

### Identification of other bacteria and possible contaminants

Apart from the bacteria that were found in culture (n = 63) in the 24 positive samples, a total of 273 bacteria, not found in culture, were identified by MetaPhlAn2 (Fig. 3). From the metagenomic sequencing of two negative controls (duplicates), we identified 10 bacterial species (Extended Data Fig. 5). In both negative extraction controls, *Propionibacerium acnes* was the most abundant species identified. Consistently, *P*. *acnes* was found in 20/24 clinical samples (Extended Data Figs 6 and 7). Moreover, we observed that the relative abundance of *P*. *acnes* in samples was negatively correlated to their total DNA concentration (Extended Data Fig. 6), in consistence with *P*. *acnes* being a contaminant in this study^17, 18^. For other species, such correlation could not be tested because of their low occurrence in samples. Beyond the 10 contaminants identified in the negative controls (found in 29 occurrences), we identified 23 putative contaminants found in 37 occurrences (Fig. 3, Supplementary Table 3), some already reported as reagents contaminants^19^. Others were more unexpected such as *Borrelia sp*. (samples 103, 104, 108 and 110) or *Rickettsia japonica* (sample 117). Still, the taxonomic assignment of the contigs did not confirm the presence of those species and manual Blastn of reads against the NCBI nr database supported that they were likely *in silico* contaminants^19, 20^. In summary, the mean percentage of reads assigned to bacteria that were considered as contaminants was 0.7% (range 0.01–5.4%). Besides, we identified 25 species that could be due to a misclassification of reads to closely related bacteria, such as in samples 184 (where *Corynebacterium striatum* was found in culture, and some metagenomic reads were identified as *Dermabacter* sp. and *Corynebacterium pyruviciproducens*), samples from patient C (19, 103, 104 where *Acinetobacter baumannii* and *Achromobacter xylosoxidans* were found in culture, and some reads were identified as from other *Acinetobacter* spp., *Achromobacter* spp., or *Advenella kashmirensis*, a bacterium close to *Achromobacter*) (Supplementary Table 3). Hence, a total of 183 bacteria not recovered in culture and not acknowledged as contaminants were identified in metagenomic sequencing. For one sample that was monomicrobial in culture (sample 46, that yielded *S*. *anginosus*), 38 other species were identified by metagenomics. Interestingly, these species appeared to be commonly found in the oropharyngeal microbiota, which was consistent with the site of the infection (mandible). In polymicrobial samples such as samples 4 and 140 (patient B), 90 and 158 (patient H), 108 and 181 (patient I), several anaerobic bacteria were identified (range 3–40, see Supplementary Table 3) in consistence with the sporadic isolation of anaerobic bacteria in culture (Table 2 and supplementary Table 3). In both samples 4 and 140 from patient B, the most abundant species was *Propionibacterium propionicum* (respective abundances of 71.5% and 43.2%) that was not found in culture. Arguments in contradiction with *P*. *propionicum* being a contaminant in these samples are that *P*. *propionicum* was not found in the negative controls, that the only *Propionibacterium* species found in other samples was *P*. *acnes*, and that the abundance of *P*. *propionicum* was high (Extended Data Fig. 7) whereas the abundance of *P*. *acnes* was low in the samples where it was identified.

### Identification of clones within species

Taking advantage of the depth of analysis of metagenomic sequencing, we addressed whether within the dominant species identified in the samples, more than one clone could be identified (see Extended Data). We assumed that in case of multiple clones within one species, the single nucleotide variants (SNVs) would be homogeneously distributed along the contigs (Extended Data Fig. 8, panels A–B) unlike when only one clone would be present (Extended Data Fig. 8, panels C-D). Accordingly, we found polyclonal populations for 29 of the 74 (39.2%) bacterial species that were tested. Among the bacteria that were found in culture and that were tested for polyclonal populations (n = 32), 8 (25%) displayed a polyclonal population: *Morganella morganii* (samples 4 and 140), *Streptococcus agalactiae* (samples 103 and 117), *Staphylococcus aureus* (samples 28 and 110), *S*. *anginosus* (sample 158) and *Pseudomonas aeruginosa* (sample 128). Moreover, we observed for *M*. *morganii* (samples 4 and 140) no mutations on the topoisomerases were found in the sample 140, while in sample 4 the Ser83Ile and Ser84Ile were found the in GyrA and ParC, respectively. This suggests that one population of *M*. *morganii* was susceptible to fluoroquinolones and the other was not. In culture though, only the fluoroquinolone resistant clone was found.

### Antibiotic resistance determinants, linkage with the host and inference of antibiotic susceptibility

A total of 151 ARDs (61 unique) were identified from the 24 samples (range 2–22, Table 3). The most frequent ARD families were beta-lactamases (n = 30), Tet(M) (n = 26), Erm (n = 18) and Dfr (n = 16). For monomicrobial samples, we assumed that the ARDs identified by metagenomics were expressed by the bacterium that was recovered in culture. Considering together (*i*) the antibiotic class the ARDs usually confer resistance to, (*ii*) the intrinsic antibiotic susceptibility profile of the species and (*iii*) the analysis of the sequence of specific genes (*gyrA*, *parC*, *rpoB*), we could infer a *in silico* susceptibility in agreement with the phenotypic susceptibility in 94.1% (111/118) cases (Fig. 4 and Supplementary Table 3), a case being defined as the susceptibility testing of one antibiotic for one sample. Of note, the six major errors (overprediction of resistance as compared to culture) originated from sample 46 where anaerobic bacteria and likely associated ARDs were found in metagenomic sequencing only. We then attempted to link the ARDs with their host through their respective depth of sequencing. Our original hypothesis was that resistant bacteria would carry at least one ARD-encoding gene copy per genome. As a consequence, one given ARD could not be sequenced less than the median depth of sequencing per contig of its host. Hence, we plotted the respective median depth of sequencing of ARDs and bacterial species (Extended Data Fig. 9). In contradiction with our hypothesis, the analysis of monomicrobial samples (in which ARDs were assumed to be carried by the identified bacteria) showed that the depth of sequencing of the contigs assigned to the pathogen could be higher than that of the ARDs (Extended Data Fig. 9). Accordingly for polymicrobial samples, we did not attempt to link ARD and their host and we separately considered the ARDs and the bacteria found in the sample (Supplementary Table 3). This way, we inferred a correct susceptibility in 76.5% (192/251) cases. Very major errors mostly occurred because some bacteria with specific resistance patterns were not detected in sequencing (Supplementary Table 3

**Table 3:** Results of the metagenomic sequencing of the 24 samples analysed in this study and antibiotic resistance determinants.

Patient	Sample number	Monomicrobial or polymicrobial	Main species identified in metagenomic sequencing (≥1% abundance)	Antibiotic resistance genes (Resfinder)	Antibiotic resistance genes (functional metagenomic studies)	GyrA	ParC	RpoB
A	2	Polymicrobial	*Staphylococcus aureus* (99.6)	*blaZ, norA*	None	WT (*S. aureus*)	WT (*S. aureus*)	D320N (*S. aureus*)
A	28	Polymicrobial	*Staphylococcus aureus* (90.3), *Finegoldia magna* (4.3), *Peptoniphilus harei* (3.7)	*ant*(*6*)*-Ia, aph*(*3′*)*-III, norA*	*tet*(*O*)	WT (*S. aureus*)	WT (*S. aureus*)	D320N (*S. aureus*)
A	66	Polymicrobial	*Staphylococcus aureus* (99.9)	*blaZ, norA*	None	WT (*S. aureus*)	WT (*S. aureus*)	D320N (*S. aureus*)
B	4	Polymicrobial	*Morganella morganii* (5.6), *Propionibacterium propionicum* (71.5), *Bacteroides fragilis* (14.2), *Prevotella bivia* (4.1), *Atopobium rimae* (3.0)	*aadA1, aph*(*3′*)*-Ia, blaDHA-1, catA1, catA2, cepA, cfxA3, dfrA1, erm*(*A*)*, erm*(*B*)*, strA, sul2, tet*(*D*)*, tet(M*)*, tet*(*Q*)	*dfr, dfr, van, tet*(*B*)			
B	140	Polymicrobial	*Morganella morganii* (14.6), *Prevotella bivia* (6.6), *Propionibacterium propionicum* (43.2), *Bacteroides fragilis* (24.4), *Atopobium rimae* (8.3), *Parvimonas* unclassified (1.2), *Dermabacter* sp HFH0086 (1.0)	*aadA1, aph*(*3′*)*-Ia, blaMOR, blaTEM-1A, catA1, catA2, cepA, cfxA3, dfrA1, dfrA14, erm*(*B*)*, QnrS1, strA, sul1, sul2, tet*(*D*)*, tet*(*M*)*, tet*(*Q*)	*dfr, dfr, tet*(*B*)*, van*			
C	19	Polymicrobial	*Acinetobacter baumanii complex* (22.1), *Streptococcus agalactiae* (73.1), *Finegoldia magna* (1.7)	*blaADC-25, blaOXA-328, erm*(*B*)*, tet*(*M*)	None	WT (*A. baumannii, S. agalactiae*)	WT (*A. baumannii, S. agalactiae*)	WT (*S. agalactiae*)
C	103	Polymicrobial	*Acinetobacter baumanii complex* (66.9), *Streptococcus agalactiae* (26.9), *Acinetobacter pittii calcoaceticus nosocomialis* (2.7), *Finegoldia magna* (1.5)	*blaADC-25, blaOXA-328, erm*(*B*)*, tet*(*M*)	None	WT (*A. baumannii, S. agalactiae*)	WT (*A. baumannii, S. agalactiae*)	WT (*S. agalactiae*)
C	104	Polymicrobial	*Acinetobacter baumanii complex* (70.1), *Streptococcus agalactiae* (24.2), *Acinetobacter pittii calcoaceticus nosocomialis* (3.9), *Corynebacterium resistens* (1.1)	*blaADC-25, blaOXA-328, tet*(*M*)	None	WT (*A. baumannii, S. agalactiae*)	WT (*A. baumannii, S. agalactiae*)	WT (*S. agalactiae*)
D	110	Monomicrobial	*Staphylococcus aureus* (98.2), *Propionibacterium acnes* (1.7)	*blaZ, norA*	None	WT	WT	WT
E	42	Polymicrobial	*Staphylococcus epidermidis* (99.1)	*aac*(*6′*)*-aph*(*2′′*)*, aph*(*3′*)*-Ia, blaZ, erm*(*C*)*, fosB, mecA*	*blaTEM*	S84Y (*S. epidermidis*)	S80F, D84Y (*S. epidermidis*)	T700S, D837E (*S. epidermidis*)
F	46	Monomicrobial	*Streptococcus anginosus* (2.2), *Mogibacterium* sp CM50 (34.5), *Olsenella uli* (13.8), *Atopobium* sp oral taxon 199 (13.0), *Peptostreptococcus stomatis* (6.9), *Parvimonas* unclassified (6.7), *Fretibacterium fastidiosum* (6.3), *Pseudoramibacter alactolyticus* (5.6), *Slackia* unclassified (3.2), *Slackia exigua* (2.2), *Alloprevotella tannerae* (2.0)	*cfxA3, lsa*(*C*)*, tet*(*M*)	*dfr, dfr, dfr, tet*(*M*)*, van*	C96Y	WT	D492A
G	59	Monomicrobial	*Streptococcus agalactiae* (99.9)	*erm*(*B*)*, tet*(*M*)	None	WT	WT	WT
G	117	Monomicrobial	*Streptococcus agalactiae* (99.9)	*erm*(*B*)*, tet*(*M*)	None	WT	WT	WT
G	136	Monomicrobial	*Streptococcus agalactiae* (99.8)	*erm*(*B*)*, tet*(*M*)*, tet*(*M*)	None	WT	WT	WT
H	90	Polymicrobial	*Olsenella* sp oral taxon 809 (55.9), *Pseudoramibacter alactolyticus* (9.6), *Atopobium* sp oral taxon 199 (8.4), *Eggerthella* unclassified (6.6), *Slackia* unclassified (5.8), *Mogibacterium* sp CM50 (5.3), *Slackia exigua* (1.8), *Peptoniphilus* sp oral taxon 375 (1.6), *Anaerococcus lactolyticus* (1.0)	*ant*(*6*)*-Ia, aph*(*3′*)*-III, erm*(*A*)*, erm*(*B*)*, erm*(*X*)*, strA, sul2, tet*(*32*)*, tet*(*M*)*, tet*(*W*)	*dfr, tet*(*O*)			
H	158	Polymicrobial	*Enterococcus faecalis* (3.8), *Streptococcus anginosus* (1.3), *Olsenella* sp oral taxon 809 (69.8), *Mogibacterium* sp CM50 (6.5), *Slackia unclassified* (3.8), *Peptoniphilus harei* (1.6), *Atopobium* sp oral taxon 199 (1.5), *Finegoldia magna* (1.4), *Pseudoramibacter alactolyticus* (1.2), *Anaerococcus lactolyticus* (1.2), *Peptoniphilus* sp oral taxon 375 (1.1), *Slackia exigua* (1.0)	*ant*(*6*)*-Ia, aph*(*3′*)*-III, cmx, erm*(*A*)*, erm*(*B*)*, erm*(*X*)*, lsa*(*A*)*, strA, strB, tet*(*M*)	*cfxA, dfr, dfr, dfr, tet*(*O*)*, tet*(*O*)			
I	108	Polymicrobial	*Peptoniphilus harei* (98.7)	None	*tet*(*M*)*, tet*(*M*)	NA	NA	NA
I	181	Polymicrobial	*Enterococcus faecalis* (62.5), *Propionimicrobium* (1.2), *Anaerococcus vaginalis* (0.5), *Peptoniphilus harei* (20.7), *Actinomyces neuii* (7.5), *Finegoldia magna* (7.1)	*aph*(*3′*)*-III, lsa*(*A*)	*dfr, blaTEM, tet*(*M*)	WT (*E. faecalis*)	WT (*E. faecalis*)	WT (*E. faecalis*)
J	121	Monomicrobial	*Staphylococcus aureus* (99.6)	*blaZ, norA*	None	WT	NA	WT
J	172	Monomicrobial	*Staphylococcus aureus* (94.6), *Micrococcus luteus* (3.1), *Propionibacterium acnes* (2.2)	*norA*	*blaTEM*	WT	NA	WT
K	128	Polymicrobial	*Klebsiella oxytoca* (74.2), *Pseudomonas aeruginosa* (0.8), *Klebsiella* unclassified (23.5)	*aac*(*3*)*-IIa, aac*(*6′*)*Ib-cr, blaCTX-M-11, blaOXA-1, blaOXY-2–8, dfrA14, fosA, oqxA, oqxB, QnrB1*,	*Putative beta-lactamase, blaTEM, vanB*	T83I (*K. oxytoca*)	S80I (*K. oxytoca*)	
L	171	Polymicrobial	*Staphylococcus aureus* (97.8), *Anaerococcus vaginalis* (1.6)	*blaZ, norA*	None	WT (*S. aureus*)	WT (*S. aureus*)	WT (*S. aureus*)
M	178	Monomicrobial	*Staphylococcus epidermidis* (99.7)	*aac*(*6′*)*-aph*(*2′′*)*, blaZ, erm*(*A*)*, fusB, mecA, spc, vat*(*B*)*, vga*(*A*)*, vga*(*B*)	None	S84F	S80Y	WT
N	184	Polymicrobial	*Enterococcus faecalis* (2.3), *Corynebacterium striatum* (4.7), *Finegoldia magna* (53.6), *Dermabacter* sp HFH0086 (13.7), *Peptoniphilus harei* (10.3), *Varibaculum cambriense* (8.6), *Anaerococcus vaginalis* (2.3), *Propionibacterium acnes* (2.0)	*erm*(*A*)*, erm*(*X*)	*tet*(*M*)*, tet*(*O*)	S83I (*F. magna*)		

WT: wild-type. NA: not assembled.

). Conversely and along with the observations with monomicrobial samples, most major errors occurred because some bacteria and ARDs were found in sequencing but not in culture. Of note, the prediction of susceptibility to fluoroquinolones (a pivotal antibiotic families for BJI treatment) was correct in 100% (24/24) samples.

### Retrospective review of the antimicrobials administered to the patients

In order to assess the potential clinical impact of the identification of bacteria by metagenomic sequencing that were not identified in culture, we retrospectively reviewed the antibiotic treatments received by the 14 patients in this study and the clinical outcome of their infection. A total of 60 different antibiotic regimens were administered, among which 6 (10.0%) were putatively not being active against bacteria found in metagenomic sequencing only (Supplementary Table 4). However as of January 2017 (median follow-up was 36 weeks, range 16–66 weeks), only one relapse was observed that involved *Enterococcus faecalis*. In this patient, the definitive treatment included amoxicillin, which is not active against some bacteria (Supplementary Table 4) found in metagenomic sequencing only. Still, the connection with the relapse remains speculative.

### Influence of downsizing the samples to 2 M paired reads

We ran the same pipeline analysis onto the 24 samples downsized at 2 M paired reads. We observed that the taxonomic distribution did not apparently change for the most abundant species (Extended Data Fig. 10), but the mean genome coverage of the pathogen(s) (found in culture) was slightly lower in the downsized group than in the full-reads group (respectively 25.0% vs. 30.2%, Student paired test p < 0.001, Extended Data Fig. 11). Also, only 86 ARDs were found after downsizing while 151 were detected before (Student paired test p < 0.001, Extended Data Fig. 11). Of note, the impact of downsizing was observed in both monomicrobial and polymicrobial samples (Extended Data Figs 12 and 13).

## Discussion

The main result of this study is that we showed that metagenomic sequencing could be a potential tool in the diagnostic of BJI. Indeed for monomicrobial infections, the pathogen was identified in 100% (8/8) samples and the antibiotic susceptibility prediction was successful in 94.1% (111/128) cases. In case of polymicrobial samples, the high abundance of several bacteria (mostly anaerobes) did occasionally prevent from the correct identification of the pathogens and their antibiotic susceptibility profiles. Accordingly, our findings support that currently, metagenomic sequencing of BJI samples could not replace conventional methods based on culture due to the limitations encountered when several bacterial species are present in the samples, but rather be performed in support.

Interestingly, metagenomic sequencing yielded in some ways more information than culture. First, metagenomic sequencing identified many more bacterial species than culture. Besides likely contaminants, some bacteria that were not detected by culture were probably true positive and may not have been targeted by the selected antibiotic regimen, as we observed in 10% cases. Second, we could identify multiple clonal populations within some species, which could differ in their susceptibility to antibiotics as we observed for fluoroquinolones in *M*. *morganii*. Sequencing multiple clones obtained from the culture of BJI samples would validate this finding and shall be considered for further studies. In all, using metagenomic data could help to tailor the antibiotic regimen for the treatment of BJI, and the added-value of clinical metagenomics in BJI should now be assessed.

However, there are several hindrances to the application of metagenomic sequencing to BJI samples. First, we could only sequence 24 out of 179 samples, due to a low amount of bacterial DNA recovered from the samples. This is the main limitation of this study as it reduced the diversity of clinical situations that we could address. Nonetheless, the samples from this study have been frozen and thawed, which decays some bacteria and releases DNA. As the DNA extraction method we used eliminates free DNA after lysing eukaryotic cells, it is likely that we could have sequenced more samples if they would not have been frozen. This said, recovering enough bacterial DNA (in terms of quantity and proportion with respect to human DNA) remains challenging. Also, the high costs of NGS (at least several hundreds of USD per sample) together with an unascertained clinical impact are obstacles to its reimbursement by health agencies. We tested the impact of a lower depth of sequencing (that could be achieved at a lower cost per sample) and showed that despite the taxonomic profiles of the bacterial populations were similar, the inference of antibiotic susceptibility was less accurate due to a lower recovery of genes involved in antibiotic resistance. Our results suggest that clinical metagenomics should indeed benefit from the highest depth of sequencing, despite the high cost. Eventually, identifying contaminants remains challenging. We used laboratory negative controls that did not includes all the putative contaminants that were identified in samples, suggesting that contamination may also occur during the sampling process. In this perspective, a clinical metagenomics negative control should be taken at the sampling stage.

Besides, our observations suggest that clinical metagenomics will soon require, as for clinical microbiology, a specific expertise combining clinical, biological and bioinformatic skills in order to infer clinically relevant results from metagenomic data. In this perspective, the development of clinical metagenomics will need the definition of quality standards, *e*.*g*. what is the sufficient genome coverage for a given bacterium to consider that its antibiotic susceptibility profile can be likely inferred. In the long term, algorithms should be built to provide clinicians with clear data and robust algorithms to support clinical decisions.

In conclusion, we showed that metagenomic sequencing of BJI samples was a potential tool to support conventional methods. In this perspective, its main limitations (DNA extraction, cost and data management) should be tackled, and the clinical benefit provided by clinical metagenomics should now be assessed in a prospective fashion.

## Material and Methods

### Samples

We initially included 179 per-operative samples recovered from 47 patients (range 1–8 samples per patient, Supplementary Table 1). All but 2 (swabs) were solid specimens. The quantity of material for each non-swab sample (n = 177) was macroscopically estimated: less than 1 mL (n = 100), from 1 to 10 mL (n = 60) and more than 10 mL (n = 17). The samples were collected from September 2015 to January 2016 in the orthopedic departments of the CRIOAc (Regional Reference Center for Complex Osteo-Orticular Infections), Lyon, France (https://www.crioac-lyon.fr) and stored at −80 °C until shipment in dry ice to the Genomic Research Laboratory in Geneva on April 13, 2016. The samples had previously been cultured on (*i*) one sheep blood agar plate (bioMérieux, Marcy l’Etoile, France) incubated under aerobic conditions at 36 ± 1 °C incubated day 1 and 2 for microbial growth, then discarded; (*ii*) two chocolate blood agar plates (bioMérieux, Marcy l′Etoile, France) kept under a 5% CO_2_-enriched aerobic atmosphere at 36 ± 1 °C: one plate was observed on day 1 and 2 then discarded; the second plate was conserved opened and read only after ten days; (*iii*) two Schaedler agar plates (bioMérieux, Marcy l′Etoile, France) incubated in anaerobic conditions: one plate was observed on day 2 and 5 then discarded; the second was opened only after 15 days and read; (*iv*) one Schaedler broth supplemented with Vitamin K1 (BD Diagnostic Systems, Le Pont-de-Claix, France) was kept 15 days in anaerobiosis and examined every day in search of a blur; if positive, Gram staining was performed and the broth was sub-cultured on agar plates (*i*.*e*., one chocolate agar and one Schaedler agar in anaerobic condition) incubated for 3 days; subculture of the broth was systematically done on day 15 even in the absence of a blur. When cultures were positive, each microorganism was identified using matrix-assisted laser desorption ionization-time of flight mass spectrometry (MALDI-TOF^®^ MS, Bruker Daltonics, Bremen, Germany) and antimicrobial susceptibility was tested according to CA-SFM recommendations (Vitek, bioMérieux, Marcy l′Etoile, France). The quantities of bacteria were visually counted up to 100 colony-forming units (CFUs) then beyond expressed as >10^2^. As true numbers were required to determine the proportion of bacteria in culture, we considered the value of 101 when >10^2^ CFUs were counted on the plate. No negative sample in culture was included in this study. A single bacteria or yeast was recovered for 104 out of 179 samples (58.1%), the remaining yielding 2 (24/179, 13.4%), 3 (26/179, 14.5%), 4 (14/179, 7.8%) or 5 (11/179, 6.1%) bacteria and yeasts. Eventually, we sequenced two negative controls (the same process as samples but without any biological material). This study involved already existing, anonymous samples for which a further use was authorized by the Ethical Committee of the Lyon University Hospital (September 25, 2014). According to the French guidelines, as the exploitation of the samples and associated data is not performed in an interventional way, the consent of the patient is not needed.

### DNA manipulations

Tissue samples were cut into small pieces on a disposable Petri dish support using a scalpel while the swabs were thoroughly vortexed in 1 mL physiological solution. DNA was extracted from 50–100 mm^3^ shredded sample using the Ultra-Deep Microbiome Prep kit (Molzym, Bremen, Germany) according to the manufacturer’s instructions (Version 2.0) for tissue samples. This method aims at decreasing the eukaryotes DNA through a differential lysis of human cells, followed by the elimination of free DNA. The concentration of bacterial and human DNA was determined by qPCR experiments as described previously^21^, using 16 S rRNA and beta-actin reference genes, respectively. The reference curves for bacterial and human DNA quantitation were generated using known concentrations of *E*. *coli* DH5α genomic DNA and human genomic DNA from the TaqMan beta-Actin Detection Reagent kit (Applied Biosystems, Framingham, MA), respectively. About 3 ng of DNA (the sum of bacterial and human DNA determined by qPCR) in a 50 µL volume were sent to Fasteris (Plan-les-Ouates, Switzerland) for DNA purification (resulting in a 10 µL purified solution) and subsequent sequencing. The Nextera XT DNA Sample Preparation Kit was used according to the Illumina (San Diego, CA) instructions except that 16 (instead of 12) PCR enrichment cycles were used. For samples with less than 3 ng total DNA in 50 µL of non-purified extracts, the volume of Nextera tagmentase was reduced from 5 to 3 µL. The libraries were sequenced in Rapid Run mode for 2 × 250 + 8 cycles on an Illumina HiSeq 2500 instrument (with a HiSeq Rapid Flow Cell v2) at Fasteris using HiSeq Rapid SBS Kit v2 and HiSeq Control Software 2.2.58. Demultiplexed fastq files were generated with CASAVA-1.8.2 from on-instrument base-calling by Real-Time Analysis (RTA) software 1.18.64.0. The Trimmomatic package^22^ was used to remove bases that correspond to the standard Illumina adapters.

### Bioinformatic methods

The quality of the reads was assessed by FastQC (http://www.bioinformatics.babraham.ac.uk/projects/fastqc/). The reads (fastq format) were processed with MetaPhlAn2 to get the taxonomic profile of the microbial community^23, 24^. MetaPhlAn2 uses a database of clade-specific marker genes, and normalizes the abundances of the species with respect to the genome size of the bacteria to which reads are assigned. In order to filter out the human reads, we used Kraken^25^ with default parameters and the –classified-out option on the miniKraken database, that embeds the genomes of bacteria, archaea and viruses, but noticeably not those of eukaryotes. Together with the seqtk subset command (https://github.com/lh3/seqtk), we were able to recover the pairs of reads that were found as from bacteria, archaea and viruses, and to assemble them using metaSPAdes^26^ with default parameters. The resulting contigs were annotated using PROKKA^27^. The genome coverage was assessed by processing the metagenomic contigs by QUAST^28^ against the reference genome of the species downloaded from the RefSeq database (http://www.ncbi.nlm.nih.gov/genome/). The total quality-filtered reads (fastq format) were mapped using Bowtie2^29^ (using the–local argument for accepting soft clipping) onto a database made of the ARDs from the ResFinder database^30^ (downloaded in July 2016) and ARDs from functional metagenomic studies^31–33^, eventually clustered at a 95% nucleic acid identity level (using CD-HIT^34^). The mapped reads were then assembled with metaSPAdes with default parameters. The open reading frames (ORFs) and the amino acid sequences were obtained by Prodigal^35^. The identification of ARDs was performed by blastp^36^ (with a 10^−30^ e-value) using the aforementioned ARD database, using a 80% amino acid identity threshold over 80% of the reference ARD sequence. To get the depth of sequencing of the bacterial species and of the ARDs in samples, we separately mapped the reads using Bowtie2 against the contigs assigned to one given species and against the ARDs identified in this sample. The depth of sequencing (expressed in × , *i*.*e*. the number of time each nucleotide was sequenced) was calculated using Samtools^37^ and BBMap (https://sourceforge.net/projects/bbmap). The single nucleotide variants (SNVs) were identified using Samtools, Freebayes^38^ and Tablet^39^.

The high cost for metagenomic sequencing is a major hindrance to its application in the clinical setting. To address this issue, multiplexing samples offers the possibility to decrease the cost but with a lower number of reads per sample. To assess the possibility of using a lower throughput for our samples, we downsized our samples to 2 M quality-filtered reads and applied the same pipeline as for full-reads samples.The same pipeline was applied to all samples after downsizing to 2 M paired reads using the seqtk sample command (https://github.com/lh3/seqtk). The figures were drawn using R v3.2.3 and the ggplot2 package^40^, with colors occasionally chosen from colorbrewer2.org. The raw reads (just adaptor-trimmed) are available under the Bioproject PRJNA382079.

### Identification of multiple clones within species

Besides the identification of species that were not recovered in culture, we tested whether more than one clone could be identified within the species. In this perspective, we considered the contigs >1000 bp and assigned them a taxonomy with Kraken. For the main species identified in the sample (species with >0.1% total bacterial reads with Kraken and that were not considered contaminants (see section above), with at least 10 contigs available, n = 74), we mapped the reads against their respective contigs and analyzed the distribution of single nucleotide variants (SNVs). For all species, we found some regions containing SNVs, but we assumed that whether more than one clone would be present within one species, the number of SNVs per contigs would be homogeneously distributed on the contigs, yielding a positive correlation between the number of SNVs per contigs and their size (Extended Data Fig. 8).

## Electronic supplementary material

Supplementary Table 1

Supplementary Table 2

Supplementary Table 3

Supplementary Table 4

Extended data
