# Supplementary material for: Clinical metagenomics of bone and joint infections: a proof of concept study
Source: Sci Rep. 2017 Aug 10;7:7718. doi: 10.1038/s41598-017-07546-5 (PMC5552814; doi:10.1038/s41598-017-07546-5)

Clinical metagenomics of bone and joint infections: a proof of concept study

Extended Data

Etienne Ruppé[1*], Vladimir Lazarevic[1], Myriam Girard[1], William Mouton[2], Tristan Ferry[3], Frédéric Laurent[2], Jacques Schrenzel[1,4]

1. Genomic Research Laboratory, Service of Infectious Diseases, Geneva University Hospitals, rue Gabrielle-Perret-Gentil 4, 1205 Geneva, Switzerland.

2. Centre International de Recherche en Infectiologie, INSERM U1111, Pathogenesis of staphylococcal infections, University of Lyon 1, Lyon, France; Department of Clinical Microbiology, Northern Hospital Group, Hospices Civils de Lyon, Lyon, France.

3. Centre International de Recherche en Infectiologie, INSERM U1111, Pathogenesis of staphylococcal infections, University of Lyon 1, Lyon, France; Infectious Diseases Department, Northern Hospital Group, Hospices Civils de Lyon, Lyon, France.

4. Bacteriology Laboratory, Service of Laboratory Medicine, Department of Genetics and Laboratory Medicine, Geneva University Hospitals, 4 rue Gabrielle-Perret-Gentil, 1205 Geneva, Switzerland.

Extended Data Fig. 1: Total quantity of bacteria obtained in culture (expressed in colony-forming units) and concentrations of bacterial (A) and human DNA (B) for 104 samples for which DNA was extracted (Supplementary Table 1). One sequenced sample is missing (sample 128) because the bacterial quantities were missing. Also, 19 and 7 samples with null bacterial and human DNA concentrations are not shown in the panel A and B, respectively. DNA concentrations in DNA extracts were determined by qPCR (see methods). The shaded grey area depicts the 95% confidence interval around the linear regression line. The X-axis is square-root transformed for visibility purposes.

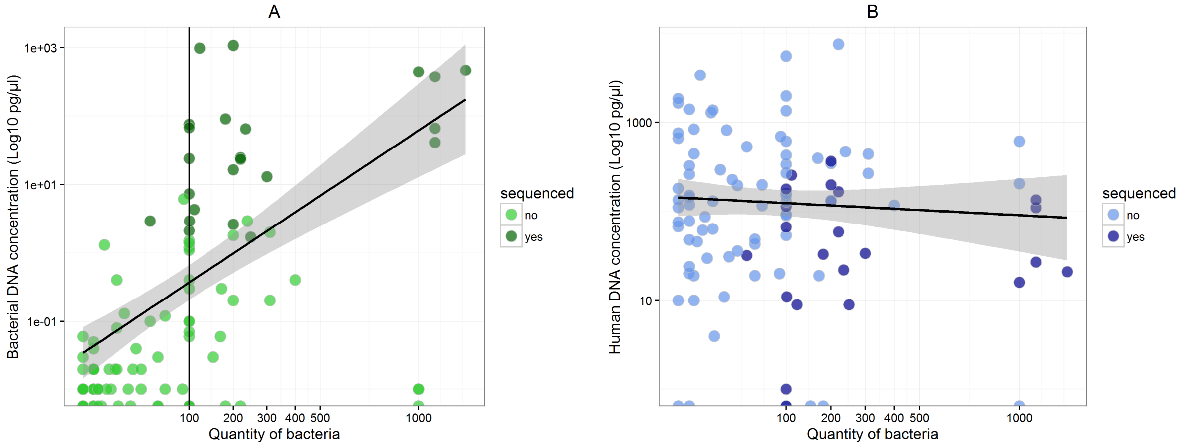

Extended Data Fig. 2: Proportion of bacterial DNA as determined by qPCR (the proportion being calculated as the percentage of bacterial DNA on the total DNA [sum of bacterial and human DNA concentrations]) on the X-axis, and proportion of the quality-filtered reads classified as bacterial, archaeal or viral by the Kraken classifier. The shaded grey area depicts the 95% confidence interval around the linear regression line.

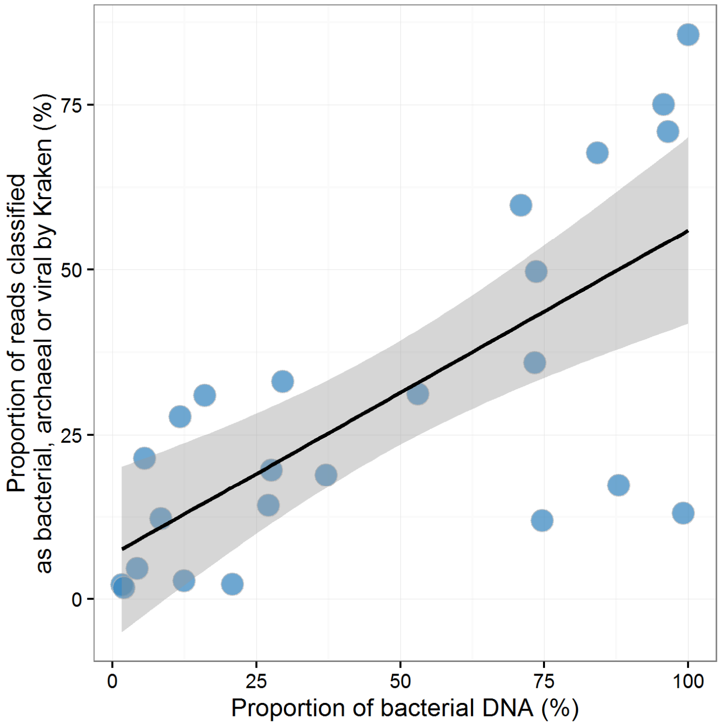

Extended Data Fig. 3: Boxplots superimposed by dots of the length of summed contigs (A), the total number of contigs (B), the total number of contigs exceeding 1000bp (C), the N50 (D), the L50 (E), the genome coverage of the bacteria species that were found in culture (F) according to the number of bacteria recovered in culture (monomicrobial or polymicrobial). The contigs were obtained by the assembly by MetaSPAdes[1] of reads classified by Kraken[2]. The parameters showed on this figure were obtained by Quast[3], using the representative genome of the species on the Genome database of the NCBI (https://www.ncbi.nlm.nih.gov/genome). *: $p<0.05$; ***: $p<0.001$; NS: not significant. The boxplot limits represents (from bottom to roof) the 25th, 50th (median) and 75th percentiles.

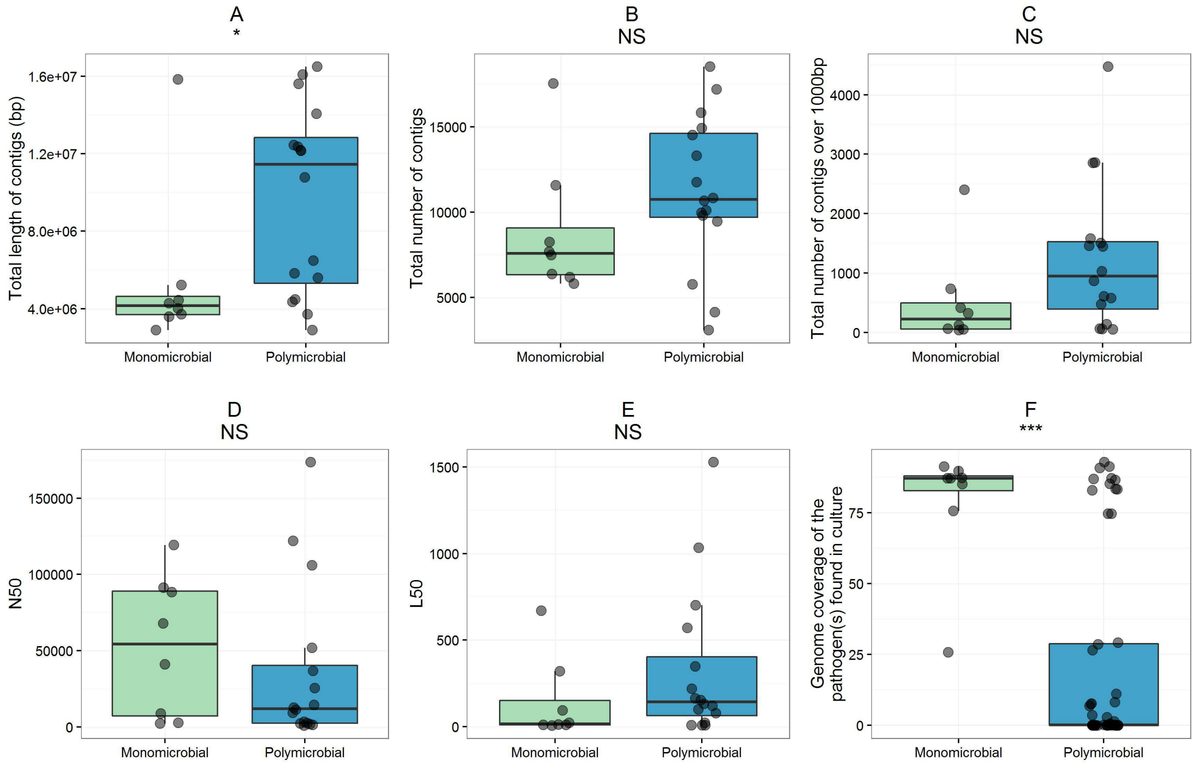

Extended Data Fig. 4: Distribution of the size of the contigs for all the samples (n=24) with respect to the results of the culture (monomicrobial [n=8] and polymicrobial [n=16]. Both Y-axis and X-axis are square-root transformed (A). Boxplot depicting the size of the contigs (Log10 transformed) with respect to the results of the culture. ***: p<0.001.

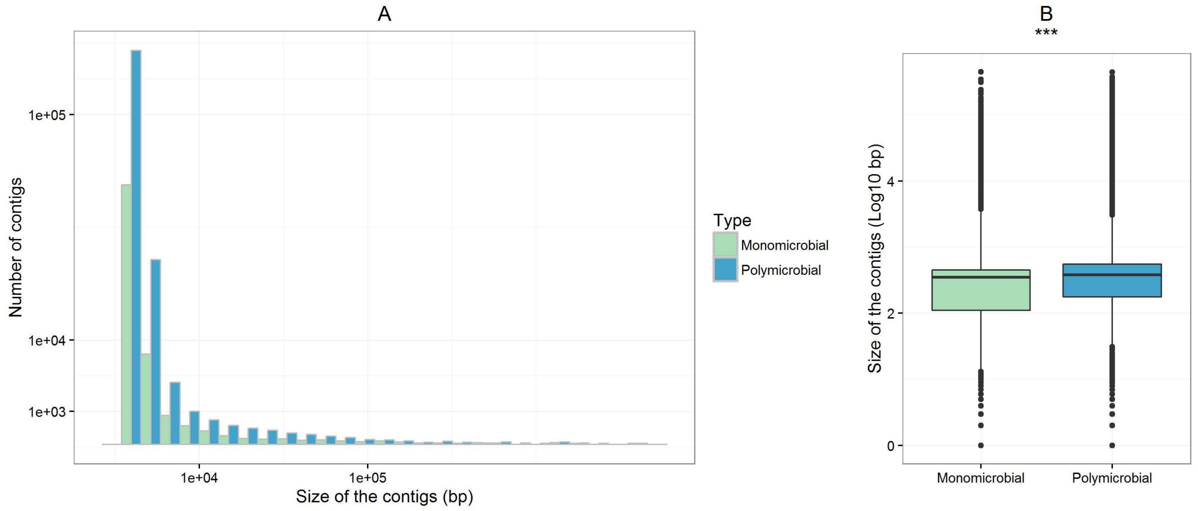

Extended Data Fig. 5: Taxonomic distribution of bacterial species found in the negative control (performed in duplicate) using MetaPhlAn2[4].

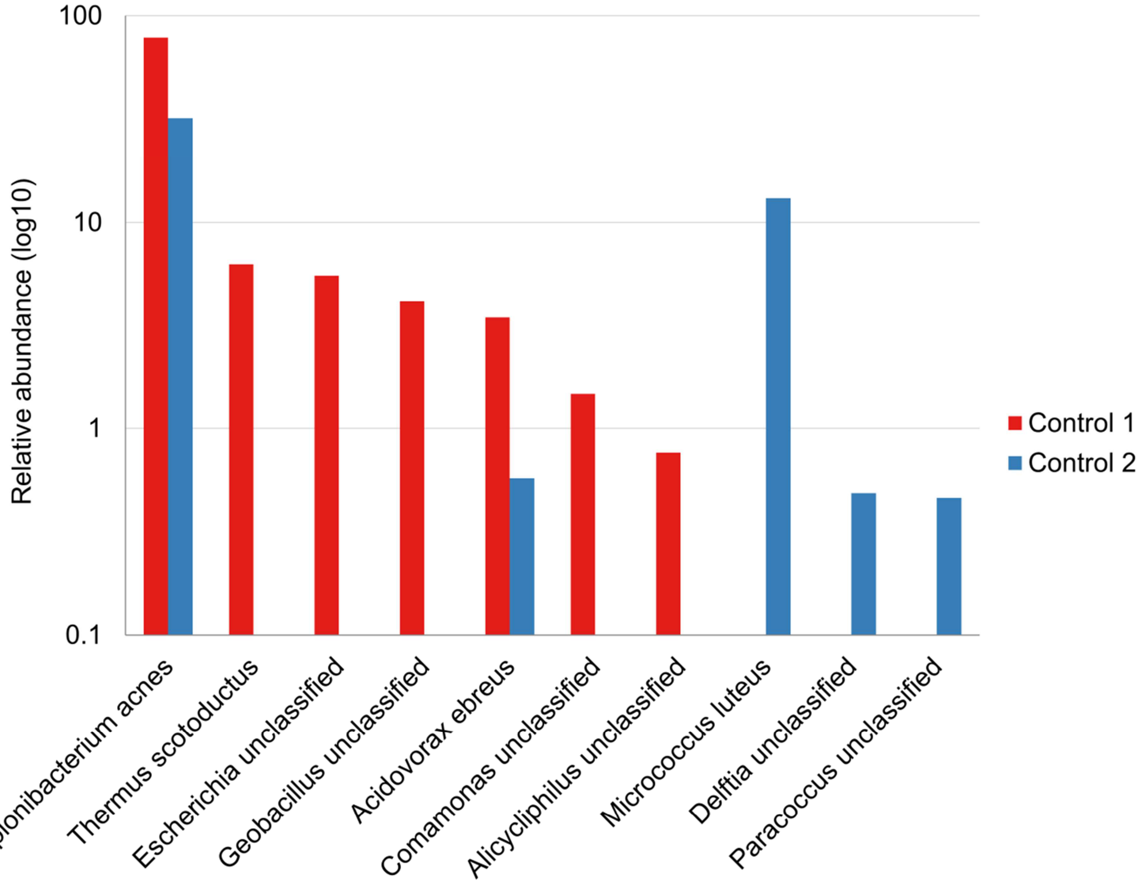

Extended Data Fig. 6: (A) Dot plot of the abundance of the species (Log10) that were considered as contaminants in this study (see Supplementary Table 3) along with the total DNA (sum of bacterial and human DNA) concentrations (pg/µL). The blue line depicts the linear regression between the abundances of the species (Log10) and the Log10 of the DNA concentrations. (B) Dot plot of the abundance of *Propionibacterium acnes* (%) along with the total DNA (sum of bacterial and human DNA) concentrations (pg/µL). Samples with an abundance of 0 are also showed. The blue line depicts the linear regression between the abundances of the species and the Log10 of the DNA concentrations.

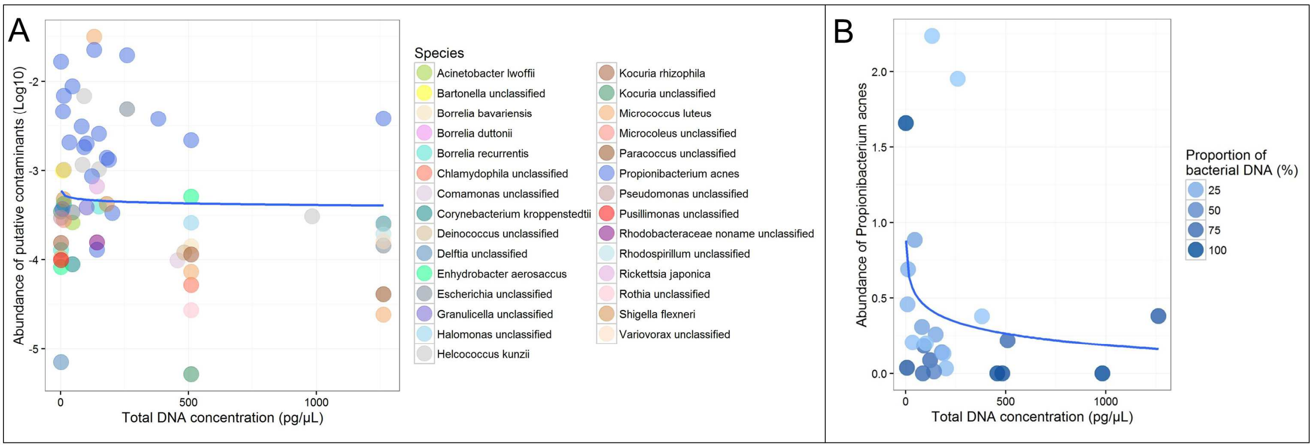

Supplement: Supplementary file 5 — Extended data [file 41598_2017_7546_MOESM5_ESM.pdf]
